# Addressing Environmental Health Inequalities

**DOI:** 10.3390/ijerph13090858

**Published:** 2016-08-27

**Authors:** Nelson Gouveia

**Affiliations:** Department of Preventive Medicine, University of Sao Paulo Medical School, Sao Paulo 01246-903, Brazil; ngouveia@usp.br; Tel.: +55-11-3061-7075

**Keywords:** environmental health, environmental risks, inequalities, proceedings

## Abstract

Environmental health inequalities refer to health hazards disproportionately or unfairly distributed among the most vulnerable social groups, which are generally the most discriminated, poor populations and minorities affected by environmental risks. Although it has been known for a long time that health and disease are socially determined, only recently has this idea been incorporated into the conceptual and practical framework for the formulation of policies and strategies regarding health. In this Special Issue of the *International Journal of Environmental Research and Public Health* (IJERPH), “Addressing Environmental Health Inequalities—Proceedings from the ISEE Conference 2015”, we incorporate nine papers that were presented at the 27th Conference of the International Society for Environmental Epidemiology (ISEE), held in Sao Paulo, Brazil, in 2015. This small collection of articles provides a brief overview of the different aspects of this topic. Addressing environmental health inequalities is important for the transformation of our reality and for changing the actual development model towards more just, democratic, and sustainable societies driven by another form of relationship between nature, economy, science, and politics.

## Editorial

Similar to the distribution of wealth and resources, in many situations we can notice that environmental health problems are unequally distributed among populations. We refer to these differences in environmental health conditions as environmental health inequalities, which can be described as health hazards disproportionately or unfairly distributed among the most vulnerable social groups, generally the most discriminated, poor populations and minorities affected by environmental risks. In other words, the main concept behind the issue of environmental health inequalities is that some population groups have a greater share of the health problems associated with environmental exposure.

Although it has been known for a long time that health and disease are socially determined, meaning they are distributed in society through strong social, economic, cultural, environmental, political and other determination processes, only recently has this idea been incorporated in the conceptual and practical framework for the formulation of policies and strategies toward health. Therefore, environmental health inequalities have recently been highly placed on the agenda of many environmental epidemiologists and other health-related professionals, in both the underdeveloped and developed world.

In this Special Issue of the *International Journal of Environmental Research and Public Health* (IJERPH), “Addressing Environmental Health Inequalities—Proceedings from the ISEE Conference 2015”, we incorporate nine papers that were presented at the 27th Conference of the International Society for Environmental Epidemiology (ISEE), held in Sao Paulo, Brazil, in 2015. These articles provide a brief overview of different aspects of this topic. 

To have an interesting introduction to the theme of environmental health, readers should start with the article by Butler [1]. He summarized the discussions held in one of the symposia at the conference about the new era called the “Anthropocene”. Recently gaining growing scientific recognition, this refers to an era in which human actions have profoundly changed the Earth’s system. This topic is bound to be prominent within future environmental epidemiology and public health.

Two other articles directly address the issue of environmental health inequalities [2,3]. The first developed an index-based approach to assess multiple burdens and benefits of differential exposure in combination with vulnerability factors that can contribute to health inequalities [2]. The second article developed indicators for mapping socioeconomically driven environmental inequalities in order to identify needs for planning interventions in a city in Germany. They identified multiple environmental burdens and hotspots of environmental inequalities related to health [3].

Three other articles are directly related to outdoor air pollution [4,5,6]. Abe and Miraglia [4] performed a health impact assessment of air pollution in São Paulo (Brazil), one of the largest megacities of the world, considering different abatement scenarios of air pollution. The air quality and health impacts of the growing use of biofuels, especially ethanol, are examined again in the context of this Brazilian city where a large proportion of the vehicle fleet runs on ethanol [5]. The third article, presented by Weaver et al. [6], observed little evidence that residential proximity to major roads, a marker of long-term exposure to traffic-related pollution, was associated with impaired cardiac function among African Americans.

Not directly discussing the health impacts of outdoor air pollution, there is an article that shows the beneficial health effects of living in communities with more vegetation [7]. This is a topic of growing importance and recognition, contributing to the increasing literature on green spaces and health pointing out that a greener city might also be a healthier city.

Lastly, Dias et al. [8] examined temporal and spatial trends in childhood asthma-related hospitalizations in Brazil and their association with social vulnerability, and Marsili et al. [9] presented a review about the health impacts of asbestos and discussed the role of epidemiological investigations in countries where asbestos is still used, suggesting an agenda for an international cooperation framework dedicated to fostering a public health response to asbestos to prevent asbestos-related diseases. 

This small collection of articles provides just a brief overview on the current knowledge on environmental health inequalities. Inequalities materialize in territories through economic investment and public policy involving many sectors and activities, such as agriculture, mining, energy production, industry, waste management, and building projects, among others. Facing this problem is one of the greatest challenges of contemporary public health. Differences in exposure to environmental risks exist everywhere, but these inequalities can be reversed when there are social movements from affected communities and other organized groups that denounce the situation and fight for their rights. Environmental and health scientists also have an important role in investigating, identifying and making these inequalities visible to decision-makers and other important players.

The combined action of researchers, citizens and social movements and organizations has the potential to reverse or at least reduce the injustices and inequalities in environmental health. These efforts will be important for the transformation of reality and the actual development of a model towards more just, democratic and sustainable societies driven by another form of relationship between nature, economy, science and politics.
